# Supplementary figures and images for: Extracellular Vesicle miR-200c Enhances Gefitinib Sensitivity in Heterogeneous EGFR-Mutant NSCLC
Source: Biomedicines. 2021 Feb 28;9(3):243. doi: 10.3390/biomedicines9030243 (PMC7997352; doi:10.3390/biomedicines9030243)

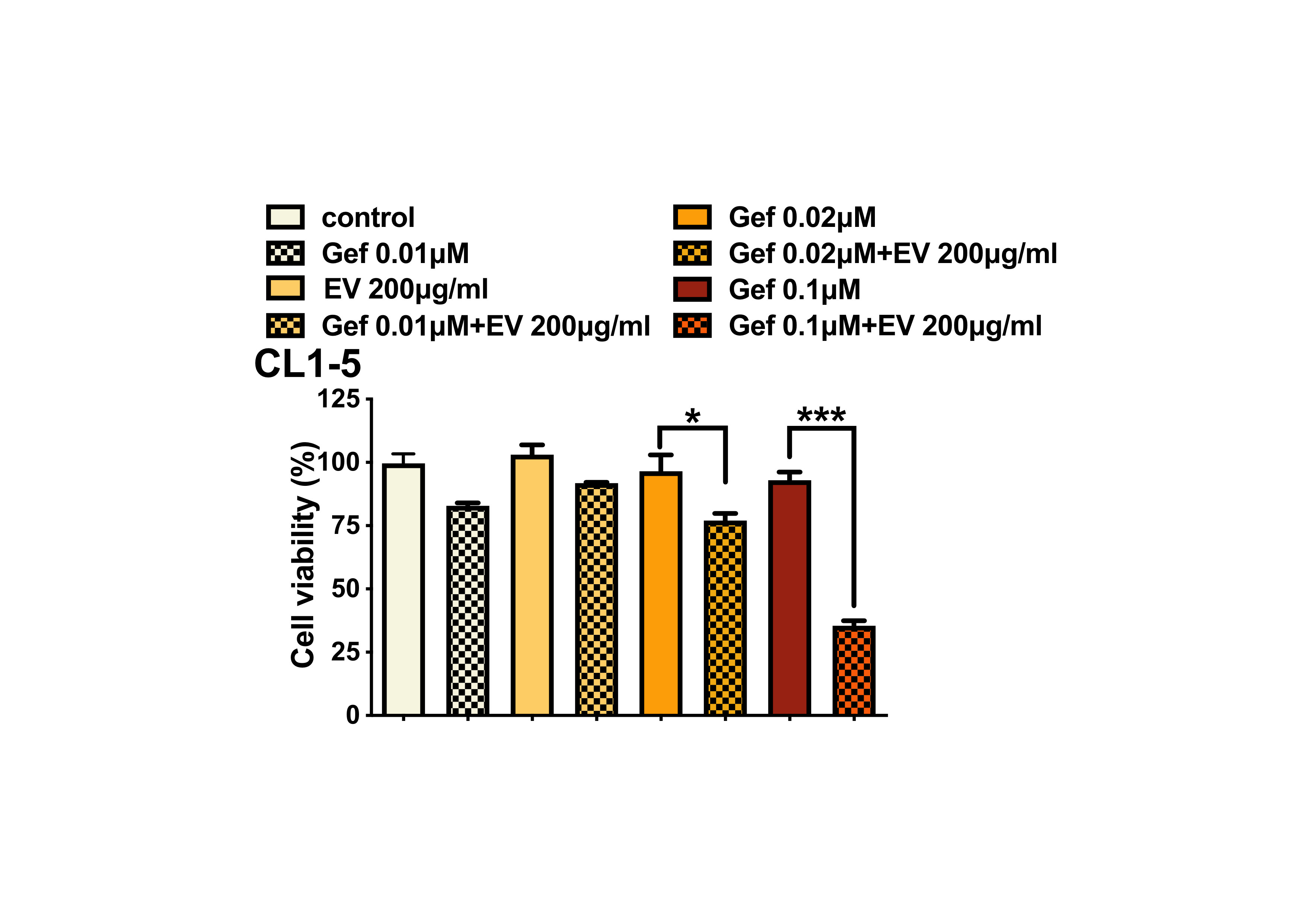

Supplement: Supplementary file 1 [file biomedicines-09-00243-s001.zip › supplement data-approval/1. supplementary figure.tif]
